# Diagnosing Gaps in the Development of Palliative and End-of-Life Care: A Qualitative Exploratory Study

**DOI:** 10.3390/ijerph17010151

**Published:** 2019-12-24

**Authors:** Helen Y. L. Chan, Diana T. F. Lee, Jean Woo

**Affiliations:** 1 The Nethersole School of Nursing, Faculty of Medicine, The Chinese University of Hong Kong, Hong Kong SAR, China; 2 CUHK Jockey Club Institute of Ageing, The Chinese University of Hong Kong, Hong Kong SAR, China

**Keywords:** healthcare system, end-of-life care, health policy, quality of care, palliative care

## Abstract

A global report found that the quality of dying in Hong Kong lagged behind that of other high-income economies. This study aims to examine the service gaps by conducting a qualitative exploratory study from multiple stakeholders’ perspectives. Purposive and snowball sampling strategies were used to maximize variation in the sample. We interviewed 131 participants, including patients, family members, health care providers, administrators, lawyers, and policy makers. The situation analysis helped identify the facilitators and barriers at individual, organizational, and socio-cultural levels that affect service development. Findings showed that awareness on palliative and end-of-life care is growing, but the existing care is limited in terms of acceptability, coverage, variation in practices, continuity, and sustainability. A number of policy, economic, socio-cultural, environmental, and legal factors were also found to hinder service development. Findings of this study demonstrated that the development of palliative and end-of-life care services involved a paradigm shift relating to society as a whole. The overarching theme is to formulate a government-led policy framework. Furthermore, a public health approach has been advocated to create a supportive environment for service development.

## 1. Introduction

Palliative and end-of-life care has been considered an ethical practice and an integral part of care for all types of chronic progressive diseases [1,2,3,4]. Literature has shown that the end-of-life care needs of patients with chronic diseases and frail older adults are poorly addressed in the current disease-centred biomedical model of care [5,6,7]. The progressive deteriorating nature of these conditions calls for a new model of care that ameliorates symptoms and promotes dignity of the end of life to counter the phenomenon of the medicalization of death [8].

The health care services in Hong Kong are renowned for their cost-effectiveness; its infant mortality rate is among the lowest and its life expectancy is highest in the world [9]. Although palliative care has been part of the health services for nearly four decades, the 2015 Quality of Death Report published by the Economist Intelligence Unit showed that the quality of end-of-life care in this city lagged behind that of many other high-income regions [10]. Out of 80 included regions, Hong Kong was ranked 22nd, which is lower than several other Asia-Pacific economies, including Taiwan (6th), Singapore (12th), Japan (14th), and South Korea (18th). The report emphasized that the ranking of Hong Kong was relatively low among the high-income regions, and the poor rating was associated with the low healthcare spending, lack of policy evaluation, inadequate capacity to deliver palliative care services, and poor community engagement related to end-of-life care services [8]. These findings were alarming to local society because these appear contradictory to the reputable and highly advanced health services in the territory.

In recent years, awareness has been growing in improving palliative and end-of-life care in local society. With over 90% of deaths occurring in hospitals, some clinical departments, such as oncology, geriatrics, and emergency departments, are seeking solutions to improve the care for seriously ill patients and their family members. Collaboration between palliative care and non-palliative care services to address the needs of these patients has been underscored owing to the huge service demand. The Hospital Authority, a statutory body that governs public hospitals, has recently formulated a strategic service framework on palliative care services to guide service development [11]. Likewise, many non-government organizations and professional organizations have conducted various programmes to promote public education or community-based end-of-life care services at their own initiative, with the support of philanthropic bodies. Given these endeavours, overhauling the existing palliative and end-of-life care services is a timely initiative. This paper reports the barriers and challenges in the macro-environment that hinder the development of palliative and end-of-life care in Hong Kong. We used the PESTEL (Political, Economic, Socio-Cultural, Technological, Environmental, and Legal) framework for situation analysis [12]. PESTEL is usually a precedent to other situation analyses for identifying specific aspects in the macro-environmental context that may exert an influence on the implementation of initiatives.

## 2. Materials and Methods

### 2.1. Study Design and Participants

A qualitative exploratory approach, through face-to-face semi-structured interviews, was used to gain a full understanding from multiple perspectives of different stakeholders toward the current end-of-life care in Hong Kong. The focus of the interviews was to explore their experience with end-of-life care and perceived factors that affect its development.

### 2.2. Sampling and Participants

Purposive and snowball sampling strategies were used to maximize variation in the sample in terms of demographic characteristics and experience related to end-of-life care. Individual interviews were conducted with care recipients, including patients, their family members, and bereaved families; individual or focus group interviews were conducted with health care providers of different ranks and disciplines from various hospitals and organizations, and any relevant roles or disciplines involved in the service development, depending on their availability.

### 2.3. Procedures

The team sent invitations via email or post to health professionals and administrators in different departments, hospitals and organizations, and to members of professional groups to invite them to participate in the study. A poster about the study was posted in public areas and social media to invite people in different capacities to join. This process was done to ensure that a heterogeneous group with different voices could be included in the sample. The interviews were conducted by the first author (H.C.) or a trained research assistant at times and places convenient to participants. Each individual interview lasted for approximately 60 min, whereas each focus group interview lasted for approximately 120 min. All participation was on a voluntary and anonymous basis. All participants completed a written consent form for inclusion before they participated in the study. The study was conducted in accordance with the Declaration of Helsinki, and the study was approved by the University Survey and Behavioural Research Ethics Committee.

### 2.4. Data Analysis

Interviews were audio-recorded with participants’ consent and transcribed verbatim to ensure an accurate record of the sharing. Data collection and analysis were conducted in an iterative process. Thematic analysis was used to identify the key issues that emerged from the qualitative data analysis [13]. First, the investigators read through the transcripts to obtain an overall picture. Then, an initial list of codes was identified on the basis of the framework of the situation analysis. The codes were compared constantly for similarities and differences to identify ambiguities and overlaps. This step facilitated the identification of persistent patterns and differences within and across the codes. The codes were collated into themes at a broad level that exhibited the latent content in the context. The process required repeated reviews and refinement. QSR NVivo version 11.0 (QSR International, Melbourne, Australia) was used to support data management.

The trustworthiness of the study was enhanced by various strategies [14]. All the audio-recordings, transcripts, and field notes following the interviews and data reconstruction products during the study process were kept, thereby providing an audit trail. The research team independently reviewed the findings to prevent idiosyncratic data interpretations. Peer debriefing with other scholars and clinicians in the field was conducted to achieve external validation. Rich and thick descriptions of each subtheme were provided to enable transferability.

## 3. Results

A total of 131 participants were interviewed between March and December 2017. They included 25 patients with different life-limiting conditions; 15 family members who were taking care of their sick relatives or had taken care their dying relatives; 50 health professionals from various disciplines; 15 frontline care staff; 15 administrators at the management levels at hospitals, care homes, and non-government organisations; and 11 participants with diverse backgrounds, such as journalists, academics, lawyers, and volunteers. Table 1 shows the demographic characteristics of the participants.

The findings were categorized on the basis of the PESTEL framework, with a detailed description as follows.

### 3.1. Political: Low Priority on the Policy Agenda

Participants generally considered the development of palliative and end-of-life care services in the local community to be fragmented with limited coverage. Many of them ascribed the underdeveloped service to the absence of a policy framework to direct the overall service development. In recent years, some government departments began to be aware of the importance of end-of-life care. However, many other social issues are pressing, such as housing and education. The priority of end-of-life care is low on the policy agenda. Several participants who were experienced in this field used the term “bottleneck” as a metaphor to describe the current situation in which the performance of the palliative and end-of-life care remained limited due to inadequate policy guidance, resulting in lose-lose results for the healthcare system, healthcare providers, and clients.

### 3.2. Economic: Lack of Consistent Funding to Support Care Services

Given that government policies specifically on the development of palliative and end-of-life care services are absent in society, the government funding for this aspect has fluctuated. Participants recalled that the funding for palliative care was among the first to be suspended during the economic recession in the past decade. Some participants also noted that the public health care funding for end-of-life care is a low priority and mainly for inpatient care in public hospitals. At present, relevant initiatives were mainly supported by charitable foundations. Given that these funding bodies avoid supporting similar projects continuously, they needed to be discontinued once the funding has ended, even if the services are beneficial for society. The one-off funding mode also affects service sustainability and staff stability. By contrast, some participants were sceptical that palliative and end-of-life care had been considered a means of decreasing the healthcare utilization and costs.

### 3.3. Socio-Cultural: Unfavourable Culture for Promoting Palliative and End-of-Life Care

The socio-cultural factors are complicated and could be further divided into several layers at the societal, familial, and professional levels.

#### 3.3.1. Denial of Death

Participants noted that death is a cultural taboo in the local community. People avoid talking about it for fear that it would attract bad luck; therefore, raising the topic for discussion can be considered ominous. Such avoidance had even diffused into daily life. For example, the number “four,” which has the same pronunciation as death, is avoided in the block numbers on an estate or in the floor number of a building. Thus, some healthcare providers were hesitant to discuss prognoses and end-of-life care with patients or their family members because they may be considered not being active in treating patients. Public education about death and dying issues are inadequate. When patients become critically ill, family members are generally emotionally unprepared because they had never thought that the patient’s condition may deteriorate. Consequently, they may think that the “sudden” health changes were due to malpractice. Complaint cases on poor communication related to end-of-life care are increasing.

#### 3.3.2. Myths about Filial Piety

Some participants maintained that the traditional belief of filial piety is also a reason that contributes to the death-denying culture. Many family members feel obliged to try every means to extend a patient’s life, regardless of the cost and consequences. Some family members thought that at least they need to do something because refusing life-sustaining treatments is deemed as giving up on the patient. By contrast, some patients and older adults who understood the limitations of medicine wished for comfort care at the end of life. They stated that their family members were the ones who felt uncomfortable with the end-of-life care discussion.

#### 3.3.3. Strong Belief in Medical Authority

Society strongly believes that medical doctors are authoritative in treatment decisions; thus paternalism prevails. Such a belief is rooted in an old Chinese saying, *“medical doctors possess parents’ hearts.”* Therefore, people generally trust that medical doctors could make the best decisions for the patients. Nevertheless, some patients and family members shared that they were confused with the incongruent advice on goal of care from different health care providers.

### 3.4. Technological: Less Alluring than Biomedical Sciences

Compared with using biomedical sciences to treat diseases, palliative and end-of-life care that highlights compassionate and humanistic care seems less alluring for career development for health professionals.

#### 3.4.1. Cure-Oriented Approach

The rapid advancement in medicine further contributes to the death-denying culture. Over the years, much of the health care resources have been invested in top-notch medical devices and advanced technology. Patients and family members were eager to search for information on various treatments, such as target therapy, immunotherapy, organ transplantation, and complementary and alternative therapies as if a cure should exist for every condition. Likewise, mortality rate is a major key performance indicator of medical services; a patient’s death appears as a failure of the healthcare team. A medical doctor stated that part of monthly departmental meetings was to review what treatments have been attempted before a patient’s death, regardless of the patient’s conditions. The focus was to rule out the possibility of premature death due to negligence, with little attention to the quality of care in the dying process.

#### 3.4.2. Lack of Professional Training and Education

The current palliative and end-of-life care service development and promotion have been highly reliant on committed people. Drawing on the experiences of participants, the value of end-of-life care services was not recognized by most health care providers. Such a problem was apparent in specific units or specialties, such as surgical departments, intensive care units, cardiac care units, paediatric units, and emergency departments, even though patients with serious illnesses accounted for a high proportion of their clients. Some participants noticed that the awareness or knowledge about the concept of palliative care or end-of-life care issues among their colleagues in the healthcare field was not better than that of laypersons. Participants noted that palliative or end-of-life care only accounted for a few hours in their pre-registration professional training or even absent for allied health professionals. By contrast, relevant on-the-job training was enrolled on a self-selective basis. In addition, the training quotas are limited; some participants learnt it by self-directed learning or attended courses or overseas exchange programs at their own expense. Thus, development was limited in terms of availability and continuity.

#### 3.4.3. Under-Researched Areas

Several end-of-life care programs were initiated in some hospitals and long-term care homes, but the practices varied. From participants’ experience, although family objection may reduce the participation rate in research, the rejection from funding bodies and ethics committees for conducting the research is the lethal cause of these incubated ideas. The funding bodies denied the value of research in this field because these programs are presumed to definitely result in significant improvement in patients’ outcomes. Moreover, the research ethics committee intended to protect patients who were mentally incompetent. Reservations on accepting proxy informed consent by family members to participate in research were noted. Empirical research to evaluate the effects of these programs could hardly be supported.

### 3.5. Environmental: Undesirable Environment for Providing Holistic Care

#### 3.5.1. Cramped Environment in Hospitals

The environment in public hospitals is generally cramped. Sometimes, the emergency department and corridors of the wards are fully occupied by patients in beds. One doctor participant used the term “battlefield” to describe the hospital environment. Another participant whose father died from a sudden and massive stroke was shocked about his unexpected death, and she was even more upset that her father was sent to the mortuary immediately after his death. She and her family members did not have the time to mourn for her father at the bedside.

Other participants also mentioned that the process of transferring deceased patients to the mortuary was dehumanizing. The trolley for carrying deceased patients was made of stainless steel; thus, it looked cold and impersonal. Hospital workers were sometimes rude when placing dead bodies onto the trolley. The mortuaries in some public hospitals also made bereaved family members miserable; for example, they are located on a lower ground level next to garbage dumps or parking lots.

#### 3.5.2. Poorly Prepared for Home Care

Some participants cautioned about the presumption that home would be a better place of end-of-life care than the ward environment. In most cases, patients’ homes were also crowded and poorly equipped. They stated examples in which patients had to lie on the floor after being discharged from the hospital or could not bathe because of limited space. Some family members were anxious when patients were discharged because they lacked caregiving skills training or the equipment and facilities for taking care of patients. Family members commonly begged doctors to postpone the date of discharge, and patients were often readmitted to the hospital shortly after discharge. Some participants also noted that inconveniences in transportation for sick people also added burden.

Challenges identified by participants for dying at home were mainly about the liability of death outside hospitals and the logistical problem of transferring a dead body using a small lift in a residential building. The police generally need to investigate the causes of deaths that occur outside a hospital to rule out mistreatment or abuse. One participant who had experienced a relative dying at home found that the police interrogation method made her feel humiliated. Some participants worried that neighbours may be superstitious if a patient died at home, affecting the property price. A health professional participant who was involved in home care service believed that dying at home was a privilege in the local context. It was considered as difficult and costly for family to arrange a medical doctor visiting the patient at home regularly to ensure there was medical attendance within 14 days before the patient’s death so that autopsy may be waived.

#### 3.5.3. Revolutionized Long-Term Care

Some residential care homes for the elderly (RCHE) had sought funding to build a single bedroom for family members to accompany a dying resident. Although some participants appreciated the private comfortable space, others stated that these rooms have been stigmatised by residents. Participants also worried that the process of a police investigation at the RCHEs may make other residents, relatives, or neighbour be sceptical about the quality of care.

### 3.6. Legal

#### 3.6.1. Uncertainty about Advance Directives (AD)

There was no specific legislation on AD in Hong Kong. Some participants said such legislation may not help because experience in overseas countries suggested that it cannot help promote its awareness and completion. Nevertheless, some participants urged for a specific law to protect healthcare teams who follow the document, as well as to protect patients’ right to self-determination in treatment decision-making. Some participants who were health professionals raised concerns about liability although the legal status of AD is currently recognised under the common law framework, whereas some were hesitant to follow the AD if the patient’s family members had not reached a consensus on the treatment decision. Another concern was the difficulty in prognostication in chronic progressive disease, thereby posing a challenge of determining the right timing for the transition from curative care into end-of-life care. For example, reservations were expressed on withholding tube feeding from a person with advanced dementia even if he had indicated advance refusal.

Some participants shared their unsuccessful experience of seeking a medical doctor to witness their process of signing an AD. They wished to complete an AD before their condition became critical. However, the doctors were resistant because they believed that they could not yet think about end-of-life care issues at that stage of the disease, and they may change their minds later on. Occasions arose in which patients completed an AD with the support of private general practitioners, although the process was rather costly and the document was not respected by public hospitals.

#### 3.6.2. Limited Powers of Attorney and Guardians

Some participants were confused by the current clinical practice of consulting family members on treatment decisions for patients because hospital guidelines stated clearly that these are medical decisions based on patients’ best interests and that family members have no legal right regarding these decisions. At present, the legal powers of guardians were limited to providing consent to medical and dental treatment in the interests of a mentally incapacitated person, according to the Mental Health Ordinance. A guardian cannot refuse treatment on a patient’s behalf if the medical team considered it to be in the patient’s best interests. By contrast, the Powers of Attorney Ordinance only allows an attorney to manage financial matters, not medical care. Some participants pointed out that the issue that treatment decision-making for the end of life is sometimes value-laden; thus whether it is in the patient’s best interests would be subject to individual interpretation.

#### 3.6.3. Absolute Duties of Ambulance Men

Participants who worked as ambulance men for the Ambulance Command under the Fire Services Department worried that the treatment refusal stated in AD is contradictory to their rescue services as stipulated by the law. They shared the feelings of powerlessness when family members pleaded with them not to proceed with resuscitation procedures because they are obliged by their assigned duties. Although situations arose in which a doctor signed the Do Not Attempt Cardiopulmonary Resuscitation (DNACPR) form to verify that a patient was terminally ill, they hesitated to follow this medical order because the form, which is a document developed by Hospital Authority, seems an internal hospital document for personnel use only.

#### 3.6.4. Legal Concerns of Dying in Place

According to the Coroners Ordinance (Cap. 504), deaths that occur outside hospital or nursing home settings should be reported to the Coroner. Participants were doubtful about the idea of dying in place even if they knew it was the patient’s wish because these reportable deaths are subject to police investigation, autopsy, and post-mortem examination, and dead bodies needed to be kept in a public mortuary for a period of time. Deaths that occur at home may be exempted from these investigations if the deceased had been diagnosed with a terminal illness or had been attended to by a medical doctor within 14 days before his or her death. However, one participant reminded that the current understanding of the term “terminally ill” does not include chronic advanced or progressive diseases.

## 4. Discussion

Palliative and end-of-life care is gaining recognition as a basic right for all who have serious illnesses [1,2,3,4,5,8]. Nevertheless, the findings of this study illustrated that the development of palliative and end-of-life care is shaped by a range of macro-environmental factors at the societal level. As noted among the top-ranked regions ranking in the Quality of Death Report, government support is the key foundation for robust development. For example, the UK government formulated the first national strategy for end-of-life care in 2008. A national palliative and end-of-life care partnership is set up that enables the health and social care sectors to continue to improve the quality of care grounded on their experience and reflection [15]. In Australia, national consensus statements were set out to identify the guiding principles and essential elements for high-quality end-of-life care [16]. The Singapore government has formulated a national palliative care strategy to guide the entire service development [17]. The Irish experience suggested that the support of policymakers is crucial for maintaining a substantial government budget for service development to widen access [18]. After reviewing the national strategies and frameworks related to palliative care of four top-ranked countries, Morrison (2018) identified the following keys to success: involving policy makers in strategy planning to overcome challenges in the infrastructure, implementing a standardized monitoring system to uphold quality evidence-based care, and maintaining an ongoing government investment to ensure sustainability [19]. Therefore, the overarching theme is to formulate a government-led policy framework that demonstrates government leadership in guiding service development.

Moreover, the study findings revealed that palliative and end-of-life care development intertwined with a range of economic, technological, environmental, and legal issues. This result is consistent with the Institute of Medicine in the report of *Dying in America: Improving Quality and Honoring Individual Preferences Near the End of Life* that various socio-cultural, economic, and health system factors hinder the quality of end-of-life care in the United States [20]. Owing to its inherent complexity, the effects of merely institutional policies and professional societies on improving the provision of palliative and end-of-life care are insignificant [19,21]. Collaboration across organizations and care sectors is imperative to ensure equitable access to quality palliative and end-of-life care and consistency in care practices [18]. This need for change is congruent with the global movement of adopting a public health approach for advocating the development of end-of-life care [22,23,24]. This holistic approach requires partnerships among government departments, public and private organizations, and communities to create a supportive environment through formulation of policy framework, revision of law, public and professional education, and re-engineering of services. One recent example in local society is that the Food and Health Bureau has just launched a public consultation on legislating ADs and making legislative amendments to facilitate the wish of dying in place [25]. This consultation underscores the importance of extending the focus beyond medical and social care sectors in the development process, with an emphasis on empowering the entire community to support changes. Sallnow and associates (2016) concluded the effects of the public health approach to end-of-life care in three aspects: practical changes to the caring process, individual attitude and understanding about death and dying issues, and building capacity in the wider community [24]. These impacts are imperative for mobilizing community resources for sustainable practice for universal access. Therefore, the public health approach will maximize the synergistic effects of the efforts of different parties in promoting palliative and end-of-life care.

We acknowledged participation bias as a limitation of this study. People who were willing to participate in this study were interested in the topic. We attempted to address this problem by inviting a wide range of people with different backgrounds and experiences with palliative and end-of-life care services by purposive sampling. During the study process, contradictory or disconfirming evidence was sought to explore conflicting accounts or viewpoints and rival explanations. This approach contributed to a comprehensive understanding of the phenomenon of interest, thereby avoiding premature closure.

## 5. Conclusions

A number of initiatives for enhancing palliative and end-of-life care have continued to proliferate in recent years in Hong Kong. However, the findings of this study showed that the immediate experience with care for seriously ill patients mostly remain suboptimal, with limitations in coverage, acceptability, continuity, and sustainability. The situation analysis uncovered that a number of political, economic, socio-cultural, technological, environmental, and legal factors were identified as hindering the further development of the service. Thus, the government urgently needs to formulate a policy framework to shape the palliative and end-of-life care and promote its development by implementing strategies using a public health approach in a broad context.

## Figures and Tables

**Table 1:** Characteristics of participants (N = 131).

		n (%)
Groups	Patients	25 (19.1)
	Family carers	15 (11.5)
	Doctors	15 (11.5)
	Nurses	16 (12.2)
	Social Worker	7 (5.3)
	Physiotherapist	7 (5.3)
	Speech Therapist	3 (2.3)
	Clinical Psychologist	2 (1.5)
	Frontline care staff	15 (11.5)
	Chaplain	3 (2.3)
	Administrators	15 (11.5)
	Others (researchers, lawyers, policymakers, journalist, volunteers)	8 (6.1)
Gender	Female	77 (58.8)
	Male	54 (41.2)
Age	<30	11 (8.4)
	31–40	29 (22.1)
	41–50	26 (19.8)
	51–60	40 (30.5)
	>60	25 (19.1)
Education	Primary or below	11 (8.4)
	Secondary	23 (17.6)
	Tertiary	97 (74.0)
